# Migrasome as a novel organelle: Biogenesis, physiological functions, and therapeutic potential

**DOI:** 10.1515/jtim-2026-0008

**Published:** 2026-02-13

**Authors:** Rumeng Tang, Ling Zhou, Jiaran Lin, Xiangyuan Zhang, Pengfei Xie, Lili Zhang, Linhua Zhao, Xiaolin Tong

**Affiliations:** Institute of Metabolic Diseases, Guang’anmen Hospital, China Academy of Chinese Medical Sciences, Beijing, China; Clinical Medical School, Chengdu University of Chinese Medicine, Chengdu, Sichuan Province, China; Graduate College, Beijing University of Chinese Medicine, Beijing, China; Department of Endocrinology, The Affiliated Hospital of Changchun University of Chinese Medicine, Changchun, Jilin Province, China

**Keywords:** migrasome, biogenesis, physiological functions, therapeutic potential, organelles

## Abstract

Migrasomes are a recently identified type of membranous organelle formed during cell migration. They are produced by migratory cells and widely distributed across various cells and tissues. Migrasomes contain abundant signaling and bioactive molecules, playing crucial roles in embryonic development, angiogenesis, material transport, mitochondrial quality control, and coagulation, as well as participating significantly in numerous pathological processes. This paper provides a detailed overview of the latest advancements in migrasome biology research, including migrasome biogenesis, physiological functions, isolation, and identification, and their roles in the onset, progression, diagnosis, and treatment of clinical diseases. In addition, we propose novel hypotheses and outline future research directions addressing current challenges and potential clinical applications of migrasomes, which may inform their utilization in future clinical diagnostics and therapeutics.

## Introduction

Migrasomes are a novel type of organelle associated with cell migration, first discovered and named in 2014 by the research team of Yu Li at Tsinghua University.^[1]^ Initially, the team observed micron-sized vesicle structures outside cells using transmission electron microscopy (TEM), describing them as pomegranate-like structures (PLS) due to their morphology. Subsequent studies confirmed that migrasome formation directly depends on the speed and persistence of cell migration, prompting their redefinition as “migrasomes.” Typically formed during cell migration, migrasomes appear at the ends or nodes of retraction fibers, with diameters ranging from approximately 0.5 to 3 micrometers.^[1]^ Migrasomes are single-membrane vesicular structures containing internal microvesicles enriched with bioactive substances, including nucleic acids, proteins, and lipids. Migrasomes are continuously generated along cell migration paths, and some detach from retraction fibers. These detached migrasomes can be taken up by neighboring cells through phagocytosis or endocytosis, or attach to the surface of other cells or the extracellular matrix (ECM), subsequently transported to specific bodily locations, thereby mediating intercellular communication.^[2]^

Migrasomes are involved in various physiological processes, including embryonic development,^[3]^ angiogenesis,^[4]^ substance transport,^[2,5]^ mitochondrial quality control,^[6]^ and blood coagulation.^[7]^ They also participate in the onset and progression of multiple diseases,including tumors,^[8,9]^ urinary system disorders,^[10,11]^ neurological diseases,^[12]^ retinal disorders,^[13]^ viral infections,^[14,15]^ and cardiovascular diseases.^[16,17]^ Moreover, they contribute to wound healing,^[18]^ regenerative medicine,^[19]^ and immune responses.^[20]^ However, the specific molecular mechanisms underlying migrasome‑mediated biological regulation and their roles in disease initiation and progression remain insufficiently defined. This paper provides a comprehensive overview of migrasome discovery, biogenesis, and known physiological functions, and summarizes current techniques for migrasome extraction, separation, and *in vivo*/*in vitro* visualization, as well as distinctions from extracellular vesicles (EVs) such as exosomes and their involvement in the occurrence, progression, diagnosis, and treatment of diverse clinical diseases. In addition, we outline prospective research directions for migrasome biology and emphasize the remaining challenges in this evolving field.

## Migrasome biogenesis

**Figure 1:**
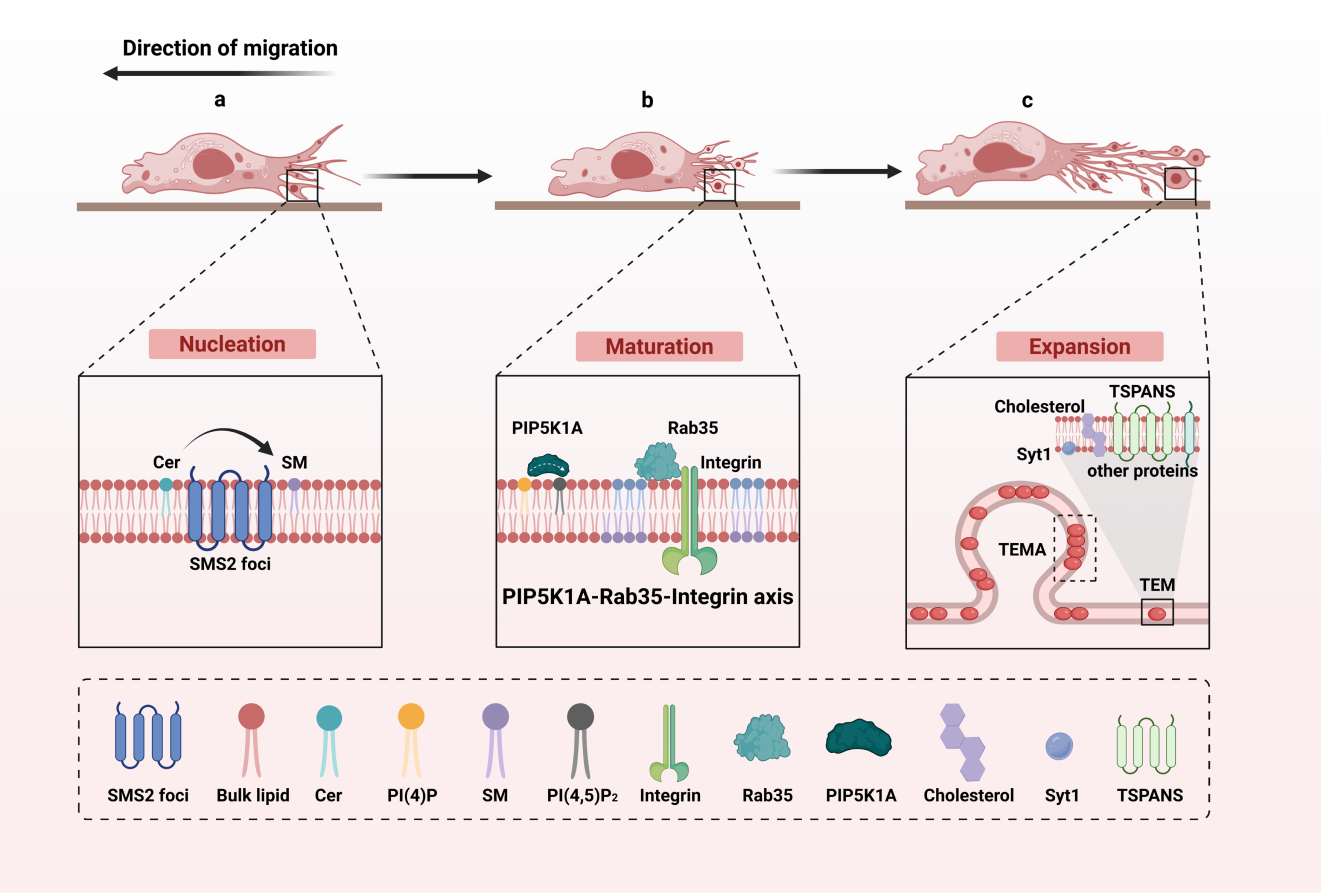
Migrasome biogenesis. The biogenesis of migrasomes is divided into three stages. (a) Nucleation: SMS2 initiates migrasome formation. (b) Maturation: PIP5K1A catalyzes the synthesis of PI (4, 5) P2, which subsequently binds to Rab35 and recruits integrins, thereby defining the maturation stage of migrasomes. (c) Expansion: The recruitment of TSPANs marks the onset of a rapid migrasome expansion phase. SMS2: Sphingomyelin synthase 2; PI (4, 5) P2: Phosphatidylinositol (4,5)-bisphosphate.

Early studies identified specialized membrane domains at retraction fiber bifurcations and termini that harbor migrasome formation sites (MFSs), spatial platforms for migrasome biogenesis.^[21]^ Once MFSs are established, migrasomes differentiate into vesicular structures through localized plasma membrane expansion. This process proceeds through three coordinated stages: nucleation, maturation, and expansion (Figure 1).^[22, 23, 24, 25]^ Migrasome formation is a highly coordinated, multi‑stage dynamic process in which nucleation, maturation, and expansion are tightly linked both spatially and temporally and mutually regulated, forming an ordered regulatory network. During persistent cell migration, the progressive rupture of retraction fibers releases mature migrasomes into the microenvironment, where they mediate intercellular communication through cargo release or endocytic uptake by adjacent cells. Importantly, migration kinetic parameters (velocity and directional persistence) directly modulate migrasome production by regulating retraction fiber stability.^[26]^

### Nucleation

Enrichment analysis of genes that specifically affect migrasome formation without altering retraction fiber structure indicates that ceramide catabolism is the most prominently enriched biological process in migrasomes.^[27]^ As a major lipid component of the cell membrane, sphingomyelin (SM) is synthesized through the conversion of ceramide, a reaction catalyzed by sphingomyelin synthase (SMS).^[28]^ Among SMS enzymes, the SMS2 isozyme is a key regulator localized to the plasma membrane, where it efficiently converts ceramide present at the plasma membrane into SM.^[29]^ Migrasomes are rich in SMS2, which actively participates in migrasome formation (Figure 1a).^[27,30]^ SMS2 specifically aggregates into SMS2 foci on the basement membrane at the leading edge of migrating cells and forms stable attachments to the matrix *via* its extracellular domain. As cells migrate forward, these anchored SMS2 foci are “left behind” at the cell rear due to their immobility relative to the cell body. Eventually, they localize to retraction fibers, becoming initiation sites for migrasome biogenesis.^[27]^ Functional experiments further confirmed that inhibition of SMS2 almost completely blocks migrasome formation, while exogenous SM supplementation effectively restores this process. These findings directly confirm that SM synthesis catalyzed by SMS2 is the core biochemical reaction for migrasome nucleation.^[27]^ Ceramide synthases (CerS), as rate-limiting enzymes in ceramide synthesis, include several family members.^[31,32]^ Knockdown experiments targeting CerS5 showed significant inhibition of migrasome formation, indicating that CerS5-mediated ceramide synthesis is a crucial source of substrates for SMS2.^[22,27]^ Notably, SM generated by SMS2 not only provides structural lipid components for migrasomes but also contributes to migrasome biogenesis by promoting the assembly of tetraspanin-enriched macrodomains (TEMAs).^[27]^

### Maturation

The conversion of ceramide to SM at nucleation sites establishes the basis for subsequent migrasome formation steps.^[27]^ Simultaneously, signaling cascades occur at these sites, transforming them into MFSs during maturation.^[21]^ Phosphatidylinositol 4, 5-bisphosphate (PI [4, 5] P_2_), a key phosphoinositide predominantly localized on the inner leaflet of the plasma membrane, plays an essential regulatory role in migrasome maturation.^[33,34]^ PI (4, 5) P_2_ is primarily synthesized by phosphatidylinositol 4-monophosphate 5-kinases (PIP5Ks).^[35]^ Prior to migrasome formation, phosphatidylinositol 4-monophosphate 5-kinase 1A (PIP5K1A) localizes to MFSs ahead of time and catalyzes the production of PI (4, 5) P_2_ at these sites.^[36]^ Subsequently, PI (4, 5) P_2_ recruits the small guanosine triphosphatase (GTPase) Rab35 to MFSs. Rab35 specifically enriches integrin α5 at MFSs by binding to the glycine-phenylalanine-phenylalanine-lysine-arginine (GFFKR) motif of integrin α5 (Figure 1b).^[36]^ In zebrafish embryos, using PIP5K1A inhibitors or peptides that disrupt Rab35-integrin α5 interactions effectively inhibits migrasome formation.^[23]^ Similarly, knocking down integrin α5 in normal rat kidney (NRK) cells suppresses migrasome generation.^[21]^ Therefore, initial PI (4, 5) P_2_ synthesis followed by the recruitment of Rab35-integrin α5 constitutes a critical maturation step during migrasome biogenesis, facilitating the transition into the expansion stage.

### Expansion

Following maturation, migrasomes expand along retraction fibers. Migrasome anchoring points preferentially stabilize at sites of strong adhesion to the ECM.^[21]^ The PI (4, 5) P_2_-Rab35-integrin signaling axis promotes the formation and stabilization of junctions by enhancing adhesion at MFSs, thus creating favorable conditions for initial expansion driven by membrane tension fluctuations.^[23,35,36]^ The expansion process depends on the recruitment and stabilization of tetraspanins (TSPANs). The mammalian Tetraspanin (TSPAN) family comprises 33 members,^[37]^ among which tetraspanin 4 (TSPAN4) is highly enriched in migrasomes, serves as a signature biomarker for migrasome identification, and plays a key role in migrasome biogenesis.^[1]^ Knocking down TSPAN 4 significantly reduces migrasome formation, whereas overexpressing TSPAN4 or other TSPAN family members promotes migrasome generation.^[24]^ During cell migration, TSPAN4 is recruited from retraction fibers onto the surface of developing migrasomes, forming dynamic puncta whose signal intensity gradually strengthens and stabilizes as migrasomes expand. Once incorporated into migrasomes, TSPAN4 does not return to retraction fibers, indicating its essential structural role in migrasome assembly.^[38]^ Migrasome membranes are rich in cholesterol and other TSPAN members, including TSPAN7 and TSPAN9.^[39]^ These proteins interact with cholesterol, membrane receptors, and adhesion molecules, forming TSPAN-enriched microdomains (TEMs). These microdomains further cluster into larger macrodomains, enhancing membrane rigidity.^[24,40]^ As retraction fibers continuously stretch, these domains expand, gradually developing into mature migrasomes (Figure 1c).^[41]^ In addition, Synaptotagmin-1 (Syt1) primes migrasome formation sites for TSPAN4-mediated stabilization by initiating their expansion.^[42]^

### Cell Migration patterns

Cell migration patterns influence migrasome formation by modulating retraction fiber dynamics. These patterns refer to the movement trajectories of cells responding to internal and external environmental signals. They directly regulate migrasome production by affecting the length, stability, and formation efficiency of retraction fibers. For example, cells migrating rapidly and linearly tend to form longer retraction fibers, thereby generating more migrasomes. In contrast, cells frequently changing direction produce narrower tails, resulting in fewer retraction fibers and thus fewer migrasomes.^[26]^ Cell migration speed influences migrasome formation by modulating the length of retraction fibers. This regulatory process likely involves molecular mechanisms such as the RhoA/ROCK signaling pathway. As the core signaling axis responsible for dynamically regulating the cytoskeleton, this pathway controls actin-myosin contractility, coordinating the severing and stabilization of retraction fibers, and consequently determining the efficiency of migrasome release. Additionally, vimentin plays a critical role in regulating cell migration behavior.^[43]^ It influences cell migration by controlling the formation of actin stress fibers or regulating lamellipodia generation.^[44,45]^ The absence of vimentin leads to reduced directional persistence and migration speed, subsequently diminishing retraction fiber formation and migrasome production. This finding underscores the decisive influence of cell migration mode on migrasome formation. Future studies should further investigate the negative regulatory mechanisms of migrasome biogenesis and provide in-depth analyses of molecular details governing active release, thereby refining the molecular regulatory network linking cell migration patterns to migrasome formation.^[26]^

## Physiological functions of migrasomes

### Maintaining mitochondrial homeostasis

**Figure 2:**
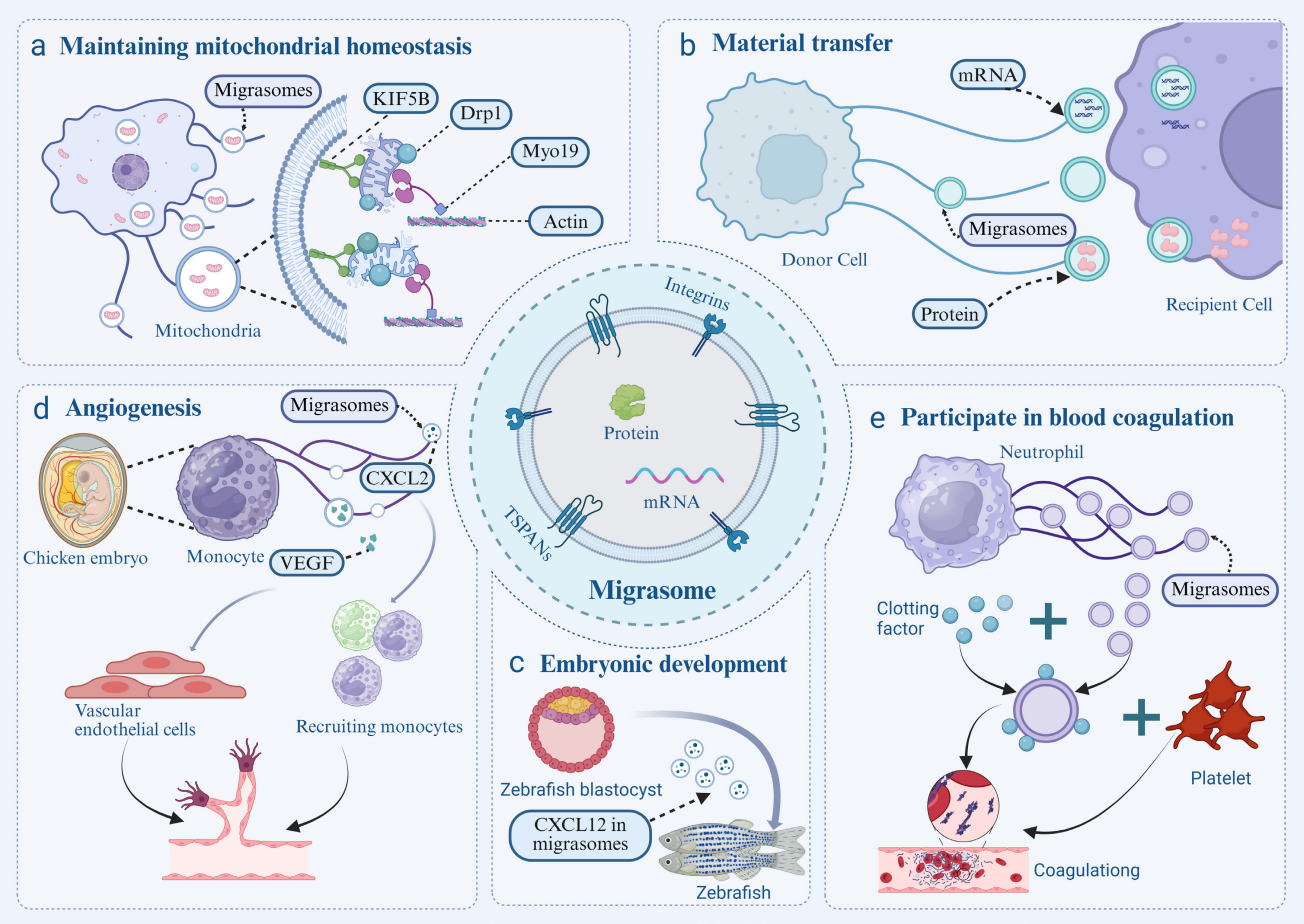
Physiological functions of migrasomes. (a) Migrasomes play a key role in sustaining mitochondrial homeostasis. Under mild mitochondrial stress, the binding affinity of KIF5B increases, promoting the transport of impaired mitochondria to the plasma membrane. Subsequently, with the involvement of Myo19, these mitochondria associate with actin at the membrane and are incorporated into migrasomes through Drp1-mediated fission prior to extracellular release. This mechanism supports mitochondrial quality control and contributes to the maintenance of cellular homeostasis. (b) Migrasomes facilitate intercellular communication through lateral material transfer. They enable the horizontal delivery of functional proteins and translationally active mRNA to recipient cells, thereby restoring the expression of specific proteins. (c) Migrasomes play a critical role in promoting embryonic development. Migrasomes derived from zebrafish blastulae contain chemokines such as CXCL12, which are essential for the proper development of zebrafish embryonic organs. (d) Migrasomes play a significant role in promoting angiogenesis. In the chorioallantoic membrane of chicken embryos, monocytes release migrasomes that are enriched with chemokines (such as CXCL12) and angiogenic factors (including VEGF). These migrasomes enhance tube formation by vascular endothelial cells and facilitate further monocyte recruitment, collectively contributing to enhanced angiogenesis. (e) Neutrophil-derived migrasomes specifically adsorb coagulation factors and accumulate at the injury site, where they activate platelets and promote the formation of hemostatic plugs through migrasome-platelet binding. This mechanism enables rapid vascular repair and effective bleeding control while preserving blood fluidity. KIF5B: kinesin family member 5B; Myo19: Myosin 19; Drp1: dynamin-related protein 1; CXCL12: C-X-C motif chemokine ligand 12; VEGF: vascular endothelial growth factor.

Mitochondria are involved in cellular aerobic respiration, and the maintenance of normal mitochondrial function is essential for cellular homeostasis. During cellular metabolism, mitochondria may experience various forms of damage, such as DNA mutations, membrane potential decline, and increased production of reactive oxygen species (ROS).^[46]^ Impaired mitochondrial function can result in metabolic disorders, cardiovascular diseases, neurological conditions, and aging.^[47]^ To maintain mitochondrial and cellular homeostasis and prevent damaged mitochondria from harming cells, defective mitochondria are specifically encapsulated in autophagosomes, which then fuse with lysosomes for degradation, a process known as mitophagy.^[48]^ Mitophagy selectively recognizes and removes damaged mitochondria to prevent further cellular harm. Jiao *et al*. demonstrated that after mild mitochondrial stress, damaged mitochondria are transported into migrasomes and subsequently exported from the cell, achieving mitochondrial quality control and maintaining cellular homeostasis. This alternative process is termed mitocytosis.^[6]^ Notably, mitocytosis occurs only under mild mitochondrial stress; severe mitochondrial stress is primarily managed through mitophagy.^[49]^ During mitocytosis, mild mitochondrial stress reduces mitochondrial binding to inward-transporting dynamin, while increasing affinity to the outward-transporting kinesin family member 5B (KIF5B). Damaged mitochondria are thus preferentially transported toward the cell periphery by KIF5B. Subsequently, these mitochondria associate with cortical actin at the plasma membrane *via* myosin 19 (Myo19), ultimately being incorporated into migrasomes through dynamin-related protein 1 (Drp1)-mediated fission (Figure 2a).^[6]^ Mitocytosis plays a crucial role in maintaining mitochondrial homeostasis, protecting cells from mitochondrial stress by discarding damaged mitochondria and thereby preserving mitochondrial membrane potential (MMP) and respiration levels. Mitocytosis thus provides the first direct link between mitochondrial health and cell migration.^[6]^

In an *in vivo* neutrophil model, neutrophils produced abundant migrasomes containing damaged mitochondria. ^[6]^ TSPAN9-knockout mice displayed reduced migrasome formation and significantly decreased MMP in neutrophils. Critically, TSPAN9 deficiency severely impaired neutrophil viability, demonstrating the necessity of mitocytosis for neutrophil survival.

### Material transfer

Following cell migration, migrasomes persist at the original location until maturation, subsequently undergoing leakage or rupture to release their vesicular contents and bioactive molecules, or becoming engulfed by neighboring cells. This indicates that migrasomal cargo can be directly transferred to recipient cells through these mechanisms, potentially constituting a novel mode of intercellular communication. ^[2]^ Migrasomes are known to contain bioactive substances, including proteins and mRNA.^[5]^ Functional proteins and translationally active mRNA molecules can be laterally transferred by migrasomes to recipient cells, restoring the expression of relevant proteins. Upon migrasome rupture and the subsequent release of various signaling molecules, they effectively mediate the biological functions of recipient cells (Figure 2b).^[5]^ However, this phenomenon has primarily been documented *in vitro*, with *in vivo* studies investigating the functional consequences of migrasome-mediated material transfer on recipient cells notably lacking. Further research is required to elucidate the physiological relevance and mechanistic details of this process in living organisms.

### Embryonic development

Migrasomes contain diverse signaling molecules, including cytokines, angiogenic factors, and chemokines.^[50]^ The regulated release of these molecules critically influences embryonic development.^[3,4]^ Researchers engineered zebrafish embryos to express migrasome markers and observed abundant migrasomes and retraction fibers *via* live-cell imaging. In TSPAN4a- and TSPAN7-knockout zebrafish, migrasome formation was reduced, resulting in developmental defects characterized by disrupted left-right asymmetry. Supplementation with exogenous migrasomes restored proper organ development, demonstrating that these developmental abnormalities directly result from migrasome deficiency rather than loss of TSPAN4a/TSPAN7 functions (Figure 2c).^[3]^ This study reveals a novel mechanism: migrasomes form membranous compartments that encapsulate signaling molecules for targeted release, thus providing precise spatiotemporal regulation of zebrafish embryonic development.^[3]^

### Angiogenesis

Vascular endothelial growth factor (VEGF), a key regulator of angiogenesis, is primarily synthesized and secreted by perivascular cells adjacent to nascent blood vessels.^[51,52]^ Secreted VEGF specifically binds to receptors on the surface of endothelial cells, triggering intracellular signaling cascades that promote neovascularization.^[53]^ Zhang *et al*. demonstrated that monocytes in the chick chorioallantoic membrane (CAM) generate migrasomes enriched with chemokines (*e.g*., CXCL12), VEGF, and transforming growth factor β-3 (TGF-β3).^[4]^ VEGF delivery *via* migrasomes establishes a pro-angiogenic niche at capillary formation sites, while migrasome-mediated CXCL12 transport recruits monocytes, creating a positive feedback loop that promotes CAM angiogenesis (Figure 2d). Monocyte depletion (*via* pharmacological or antibody approaches) or suppression of migrasome biogenesis (through TSPAN4 knockdown) severely impairs capillary neogenesis. Exogenous migrasome supplementation rescues defective capillary formation.^[4]^ These findings establish migrasomes as spatiotemporal signaling hubs: Their membrane-bound compartments package molecules for targeted release, coordinating physiological functions during angiogenesis.

### Participate in blood coagulation

Neutrophil-derived migrasomes in circulation are critical components of the hemostatic system.^[7]^ Blood from both humans and mice contains abundant neutrophil-derived migrasomes that rapidly adsorb coagulation factors and accumulate at injury sites, triggering platelet activation through exposure of surface phosphatidylserine, thus amplifying thrombin generation.^[7,54]^ Activated platelets bind migrasomes and assemble porous hemostatic plugs, maintaining blood flow while sealing damaged vessels to prevent hemorrhage. To clarify the direct role of migrasomes in hemostasis, researchers used neutrophil-depleted mice in tail-tip bleeding assays. Neutrophil depletion prolonged bleeding time, an effect reversed by exogenous neutrophil-derived migrasomes (Figure 2e).^[7]^ This study establishes migrasomes as essential hemostatic regulators, revealing a novel mechanism for coagulation control and wound healing.

## Therapeutic potential of migrasomes

**Figure 3:**
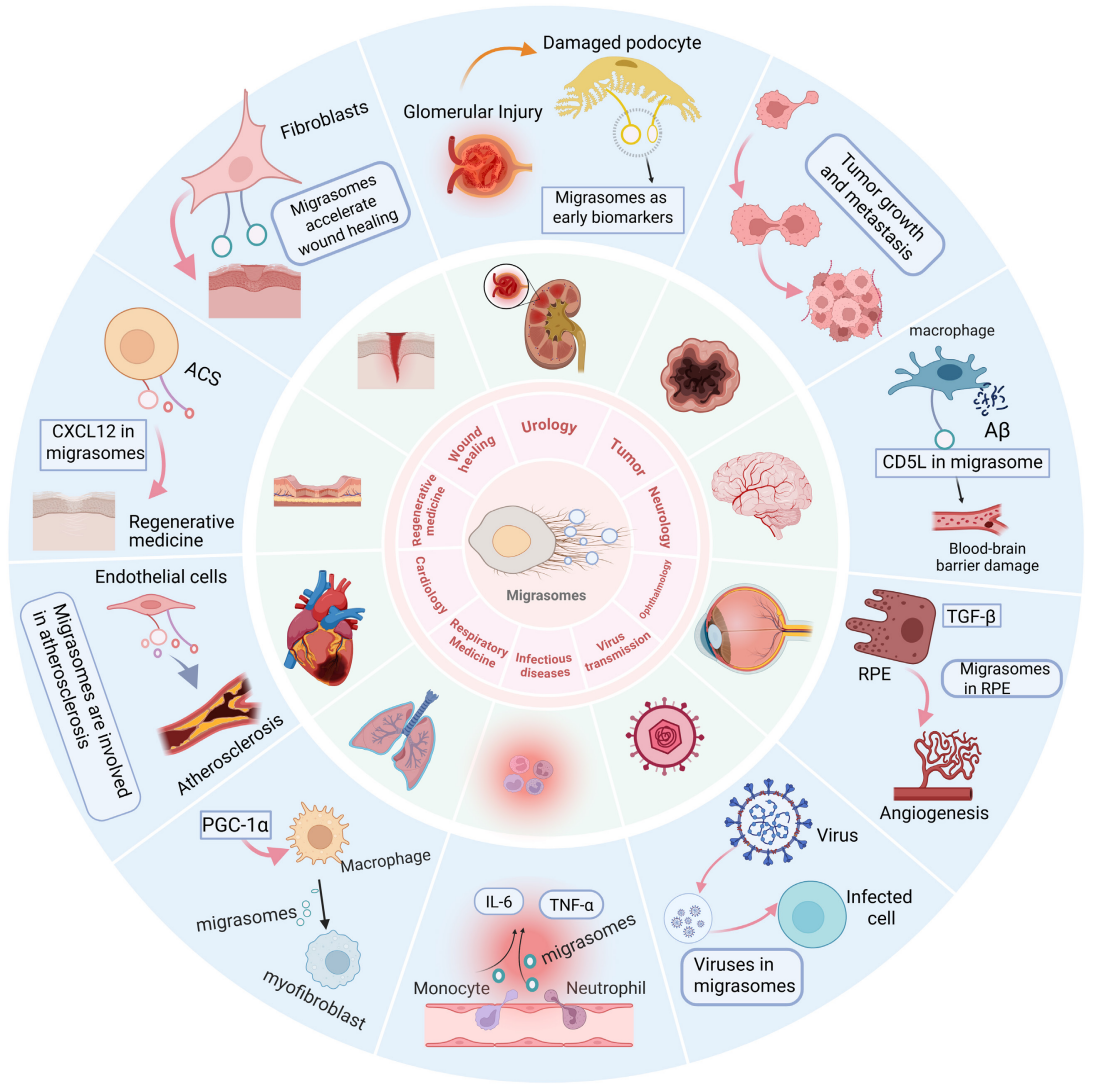
Therapeutic potential of migrasomes. Migrasomes contribute to the pathogenesis of various diseases, including urological disorders, tumors, neurological conditions, ophthalmic pathologies, viral infections, respiratory diseases, and cardiovascular diseases. They also function in wound healing, regenerative medicine, and the progression of infectious diseases.

As understanding of migrasomes’ physiological functions deepens, research into their roles in various diseases continues to grow. This study summarizes current clinical diseases associated with migrasomes (Figure 3 and Table 1). Clinical research on migrasomes primarily focuses on three aspects. First, identifying key genes related to migrasome formation in diseases, which may suggest a link between migrasomes and pathology, though independent effects of these genes cannot be excluded. For example, TSPAN4, a migrasome marker, exhibits significantly elevated expression in a mouse model of myocardial infarction, implying a potential role for migrasomes in atherosclerosis.^[16]^ However, whether migrasomes directly exacerbate this condition requires further investigation. Second, analyzing associations between migrasomes and diseases to support their potential application as diagnostic or prognostic biomarkers, such as migrasomes serving as potential early diagnostic markers for kidney injury.^[10]^ Third, migrasomes directly contribute to disease progression, such as promoting viral dissemination^[38,55]^ and tumor metastasis.^[56, 57, 58]^

**Table 1:** Mechanism of migrasome involvement in different diseases

Category	Disease	Cell types producing migrasome	clinical samples involved	In/ex vivo model sample involved	Mechanism	Effect	References
Urology	Nephropathy	podocyte	Human patient urine samples	C57BL/6 mice urine sample	Podocytes secrete more migrasomes when damaged	Migrasomes as potential diagnostic markers for early kidney injury	[10,11]
Tumor	Hepatocellular carcinoma	Hepatocellular carcinoma cells	Human hepatocellular carcinoma cell tissue	NA	CD151 upregulation enhances migrasome biogenesis	VEGF-rich migrasomes enhance hepatocellular carcinoma cell migration	[56]
	Glioblastoma	GBM cells	NA	NA	TSPAN4 drives glioblastoma progression via EGFR interaction	Migrasomes promote the proliferation and invasion of GBM cells	[58]
	Bone metastasis	Tumor cells	NA	BALB/c mice	Tumor cells activate osteoclasts via migrasome-mediated mRNA delivery, promoting PMNs formation	Bone metastasis promoted by stimulation of osteoclasts to secrete acidic substances	[8]
Neurology	Cerebral amyloid angiopathy	Macrophage	Human patient skin autopsy, Human plasma	C57BL/6 mice brain tissue	CD5L-enriched migrasomes impair complement resistance and disrupt the blood-brain barrier	Migrasomes promote Aβ deposition and brain injury	[70]
	Traumatic brain injury	Neutrophils	NA	C57BL/6 mice head cortex	Migrasomes boost neuro-immune surveillance via enhanced cell-to-cell communication	Migrasomes serve as critical signaling hubs between peripheral and central nervous system immune cells	[73]
	Acute ischemic stroke	Microglia/macrophages	Human patient brain tissue	C57BL/6 mice brain tissue	Migrasomes derived from microglia/macrophages exacerbate neuronal damage	Migrasomes exacerbate acute ischemic stroke	[12]
Ophthalmology	Proliferative vitroretinopathy	retinal pigmented epithelium cells	Human PVR membranes and normal donated retina, vitreous bodies	Rat	The migrasomes of RPE are induced through the TGF-β1/Smad2/3 pathway	Increases the migration and proliferation of RPE to induce PVR	[13]
Virus transmission	Chikungunya virus infection	Hela cells	NA	NA	The infected cells release migrasomes encapsulating virions	Promoting virus infection in the mode of cell-to-cell spread by migrasomes	[38]
	Mpox virus infection	HeLa and Huh7.5.1 cells	NA	NA	Migrasomes encapsulate virus particles within them	Migrasomes boost viral transmission efficiency and drug resistance	[15]
	HSV-2 infection	HaCaT and CHO cells	NA	C57BL/6 mice intestinal tissues	Migrasomes contain viruses	Migrasomes promote virus transmission	[55]
Infectious diseases	inflammation	Monocytes and neutrophils	NA	C57BL/6 mice plasma	Migrasomes deliver inflammatory factors to promote local inflammatory cascade reactions	Migrasomes participate in early-stage immune responses to infection	[20, 52]
Respiratory Medicine	Sepsis-associated pulmonary fibrosis	Myofibroblast and macrophage	NA	C57BL/6 mice lung tissue	PGC-1α enhances migrasome formation to drive pulmonary fibrosis	Migrasomes drive SAPF fibrosis via MMT	[84]
Cardiology	atherosclerosis	Endothelial cells	Human atherosclerotic tissue	C57BL/6 mice aorta	Endothelial-derived migrasomes drive macrophage M1 polarization	Migrasomes are involved in the process of atherosclerosis	[17]
	myocardial ischaemia–reperfusion injury	HUVEC cells	NA	C57BL/6 mice heart tissue	Migrasomes selectively remove damaged mitochondria by mitocytosis	Providing mitochondrial quality control to improve cardiac dysfunction	[85]
Regenerative medicine	-	Adipose-derived stem cells and macrophage	NA	C57BL/6 mice adipose tissue	ASCs promote stem cell migration via CXCL12-enriched migrasomes activating CXCR4/RhoA signaling	Migrasomes recruit stem cells to promote tissue regeneration	[19, 88]
Wound healing	-	Fibroblast and HaCaT cells	human skin	C57BL/6 mice skin	Young fibroblast-derived migrasomes effectively reverse aged keratinocyte senescence	Migrasomes accelerate wound healing	[18]

CD151: Cluster of Differentiation 151; VEGF: Vascular Endothelial Growth Factor; TSPAN: Tetraspanin; GBM: Glioblastoma; CDC: Complement-dependent cytotoxicity; CAA: Cerebral Amyloid Angiopathy; PVR: Proliferative Vitreoretinopathy; RPE: Retinal Pigment Epithelium; TGF-β1: transforming growth factor-β1; Mpox: monkeypox; HSV: herpes simplex virus; PGC-1α: peroxisome proliferator-activated receptor gamma coactivator 1-Alpha; SAPF: subacute pulmonary fibrosis; MMT: mesenchymal-mesenchymal transition; ASCs: adipose-derived stem cells; PMNs: premetastatic niches.

### Urology

Podocyte injury is the central mechanism underlying the pathogenesis of kidney diseases.^[59]^ Podocyte injury reduces the glomerular filtration rate and leads to proteinuria.^[60,61]^ Podocytes are terminally differentiated cells unable to proliferate or regenerate. Compared with other kidney cells, damaged podocytes display greater migratory activity under disease conditions.^[62]^ Podocyte-derived migrasomes have been detected in the urine of mice with kidney injury, appearing prior to elevated urinary microalbumin. Thus, urinary migrasomes could serve as a non-invasive diagnostic indicator for podocyte damage.^[10]^ Clinical studies have also confirmed that migrasomes derived from podocytes in the urine of patients with kidney disease are associated with kidney injury,^[10,11]^ highlighting their potential use in nephropathy diagnosis and treatment. Although these studies directly link migrasomes and podocyte injury, the causal relationship between migrasome formation and disease progression has yet to be fully established.

Rac1, a member of the Rho family of small GTPases, regulates cellular signaling, migration, and inflammatory responses. Podocyte injury is characterized by aberrant activation of the Rac1 signaling pathway. Inhibition of Rac1 preserves podocyte structural and functional integrity.^[63]^ Liu *et al*. demonstrated a significant negative correlation between Rac1 inhibitor dosage and urinary migrasome quantity in injured podocyte models. This finding suggests that the protective effects of Rac1 inhibition on podocytes may be mediated through regulating migrasome release.^[10]^ These findings indicate a potential causal relationship between migrasomes and renal injury, yet the possibility that increased migrasome release is merely an accompanying phenomenon of cellular damage cannot be excluded. Current evidence primarily stems from observational studies utilizing mouse disease models and patient urine samples. There remains a lack of *in vivo* causal validation experiments, where migrasome biogenesis is specifically modulated to directly observe subsequent renal outcomes. Moreover, the sensitivity, specificity, and predictive value of urinary migrasomes as biomarkers, compared to established clinical gold standards such as eGFR and UACR, have not been validated in large-scale prospective cohort studies. Furthermore, the isolation and quantification of urinary migrasomes lack standardized methodologies, and challenges concerning their stability and assay reproducibility pose major technical barriers to clinical translation. Given the correlation between migrasome release and the extent of podocyte injury, targeted interventions against migrasome formation may represent a novel therapeutic strategy for kidney diseases.

### Tumor

Migrasomes are widely present in highly migratory tumor cells and play a significant role in tumor metastasis. Wang *et al*. found abundant programmed death-ligand 1 (PD-L1) at the trailing edges of migrating tumor cells. After internalization by tumor cells, PD-L1-rich migrasomes can upregulate PD-L1 expression. Meanwhile, they promote tumor migration by secreting chemokines, becoming a key component of tumor metastasis mechanisms.^[9]^ Premetastatic niches (PMNs) are essential for tumor metastasis and are characterized by immune suppression, inflammatory responses, and angiogenesis.^[64,65]^ In bone metastasis, osteoclasts play a central role in forming PMNs.^[66]^ Studies have shown that tumor cells can deliver mRNA to osteoclast precursors *via* migrasomes, inducing their abnormal differentiation into osteoclasts and promoting pre-metastatic niche (PMN) formation.^[8]^ This suggests that inhibiting migrasome formation could provide a new strategy for the early prevention of tumor bone metastasis. However, this discovery is based on *in vitro* co-culture and animal models, and it is still necessary to prove whether blocking this pathway under *in vivo* conditions can completely inhibit the formation of metastatic foci. Nevertheless, further investigation is required to fully clarify the mechanistic roles of migrasomes.

The expression of cluster of differentiation 151 (CD151) in cancer cells is essential for migrasome biogenesis and may be utilized as a biomarker. Elevated CD151 expression facilitates migrasome formation, enhancing the invasiveness and angiogenic capacity of hepatocellular carcinoma (HCC) cells, thus promoting primary HCC progression.^[56]^ Further studies revealed that integrin alpha-5 (ITGA5) is upregulated in HCC tissues and correlates with poor clinical prognosis. Knockdown of ITGA5 significantly suppresses the proliferation, migration, and invasion of HCC cells, whereas its overexpression worsens malignant phenotypes.^[57]^

As a biomarker and critical mediator of migrasome formation, TSPAN4 is frequently dysregulated across multiple tumor types.^[67]^ Previous studies showed that TSPAN4 promotes glioblastoma (GBM) progression through interaction with epidermal growth factor receptor (EGFR)^[58]^ and modulates immune cell infiltration within the tumor microenvironment.^[67]^ Migrasomes facilitate immune evasion, thereby supporting tumor invasion and metastatic dissemination. Consequently, targeting migrasome biogenesis represents a promising therapeutic strategy for tumor suppression. However, given its potential interference with physiological functions in normal cells, future research should prioritize developing targeted approaches to selectively inhibit migrasome production in tumor cells.

### Neurology

Cerebral amyloid angiopathy (CAA) is a common neurodegenerative disorder characterized by β-amyloid (Aβ) deposition in small intracranial vessels.^[68]^ Predominantly affecting the elderly, CAA is an important cause of cognitive decline and intracerebral hemorrhage in this population.^[69]^ Hu *et al*. were the first to identify macrophage-derived migrasomes in CAA. Their findings demonstrated that after macrophages phagocytose Aβ, migrasomes enriched with CD5 antigen like (CD5L) are generated, exhibiting vascular adhesiveness. CD5L accumulation attenuates the host’s resistance to complement activation, resulting in complement-dependent cytotoxicity, disruption of the blood-brain barrier (BBB), and subsequent exacerbation of Aβ deposition and cerebral injury.^[70]^ Given the established association between migrasomes and CAA, migrasomes represent promising candidate biomarkers for the clinical detection of this condition. In central nervous system (CNS) disorders, the BBB poses a significant challenge to identifying early peripheral biochemical alterations.^[71]^ However, brain-resident macrophages can produce migrasomes associated with neuropathology. Future research should focus on detecting migrasome-related biomarkers in cerebrospinal fluid to provide targeted molecular evidence supporting the early diagnosis of CNS diseases such as CAA.

Traumatic brain injury (TBI) results from external mechanical forces acting directly on brain tissue. Pathology begins with primary structural damage and evolves into complex secondary injury cascades, causing transient or persistent neurological deficits.^[72]^ In a murine TBI model, significant neutrophil infiltration was observed surrounding the lesion. Recent studies suggest that neutrophils directly communicate with immune cells in the CNS by generating migrasomes. These migrasomes enhance neuroimmune surveillance responses,^[73]^ providing novel therapeutic insights for TBI treatment.

Pathological accumulation of migrasomes has been confirmed in the brain tissue of acute ischemic stroke (AIS) patients.^[12]^ A high-salt diet induces excessive migrasome production by microglia/macrophages, exacerbating neuronal damage and representing a novel pathological mechanism in AIS.^[12]^ Notably, migrasomes derived from bone marrow mesenchymal stem cells improve macrophage phagocytic capacity against bacteria, reducing post-AIS pneumonia.^[74]^ These findings collectively demonstrate the dual role of migrasomes in AIS pathogenesis.

### Ophthalmology

Proliferative vitreoretinopathy (PVR) is a vision-threatening disorder characterized by activation of retinal pigmented epithelium (RPE) cells *via* transforming growth factor β (TGF-β) cytokines, causing retinal detachment and irreversible blindness.^[75]^ PVR treatment remains challenging. Current pharmacological interventions are mainly prophylactic, with uncertain efficacy and potential adverse effects.^[76]^ Although surgery remains the primary treatment, it is invasive, complex, and requires substantial expertise and advanced equipment. Wu *et al*. reported elevated TSPAN4 expression, a migrasome marker, in clinical PVR specimens.^[13]^ Further studies revealed that TGF-β1 activation upregulates TSPAN4 expression and promotes migrasome production in rat RPE cells. Mechanistically, the TGF-β1/Smad2/3 pathway mediates both TSPAN4 expression and migrasome formation in RPE cells. Therapeutically, silencing TSPAN4 reduces RPE cell fibrogenesis and significantly suppresses epiretinal membrane formation.^[13]^ These findings underscore the critical role of migrasomes in PVR progression. Targeting TSPAN4 or inhibiting migrasome biogenesis may thus represent a novel therapeutic strategy for PVR. The formation of pathological retinal neovascularization (RNV) is a key driver of retinal diseases, stemming from an imbalance between angiogenic stimulators and inhibitors.^[77]^ Although there are currently no studies directly reporting the association between migrasomes and RNV, as potential carriers of angiogenesis, migrasomes may be involved in the disease progression of RNV.

### Virus transmission

Migrasomes are critical mediators of intercellular viral transmission.^[14]^ Upon chikungunya virus (CHIKV) infection, viral nonstructural protein 1 (nsP1) activates PIP5K1A, enhancing PI (4, 5) P_2_ synthesis and modulating actin polymerization dynamics. This induces migrasome biogenesis and increases cellular motility, promoting viral spread. This represents the first evidence that migrasomes facilitate viral dissemination.^[38]^ Lv *et al*. confirmed by TEM that migrasomes encapsulate intact mpox virus particles. Mpox virus infection notably upregulates migrasome production, enabling stealth viral transport.^[15]^ Research indicates that the antiviral drug tecovirimat/ST-246, which targets poxvirus, failed to significantly inhibit virus-induced migrasome formation, likely due to migrasomes’ greater structural stability compared to typical EVs.^[78]^ Consequently, migrasomes may function as protective compartments, allowing viruses to evade common antiviral treatments and enhancing transmission efficiency and drug resistance. Additional studies identified virus-containing migrasomes in HSV-2-infected cells and murine tissues.^[55]^ These migrasomes efficiently transmit viruses to uninfected cells, initiating new infection cycles. Migrasome-mediated viral transmission occurs at significantly higher efficiency than conventional exocytosis. Xu *et al*. demonstrated that dasabuvir suppresses migrasome formation during vaccinia virus (VACV) infection, inhibiting the propagation of extracellular enveloped viruses (EEVs).^[79]^ Therefore, targeting virus-induced migrasome formation represents a promising strategy for novel antiviral therapeutics and warrants further investigation.

### Infectious diseases

During infection and inflammation, neutrophils, acting as initial responders, are rapidly mobilized.^[80,81]^ Migrasome production by neutrophils increases dramatically, suggesting migrasome generation is integral to the immune response during infection or inflammation.^[4]^ Upon lipopolysaccharide (LPS) stimulation, monocytes utilize a migrasome-dependent pathway to secrete inflammatory cytokines, achieving substantially higher release compared to classical exocytosis.^[74]^ These cytokine-loaded migrasomes rapidly accumulate at inflammation sites, forming localized signaling centers. This highlights migrasomes as a novel cytokine delivery mechanism and potential therapeutic targets for immunomodulation and inflammatory disease treatment. Additionally, migrasome-deficient mice exhibit reduced acute inflammation and improved survival after toxin challenge, further supporting the role of migrasomes in early immune responses.^[20]^ Consequently, migrasomes represent promising therapeutic targets and early diagnostic markers for related pathologies.

### Respiratory medicine

Sepsis-associated pulmonary fibrosis (SAPF) is a critical pathology in acute respiratory distress syndrome induced by sepsis.^[82,83]^ Peng *et al*. demonstrated that migrasomes mediate macrophage-to-myofibroblast transition (MMT) in LPS-induced SAPF mouse models and fibroblast-macrophage co-culture systems.^[84]^ PGC-1α promotes migrasome biogenesis, facilitating profibrotic signaling and driving fibrosis in SAPF. This study identifies migrasomes as potential therapeutic targets for SAPF.^[84]^

### Cardiology

Migrasomes regulate cardiovascular diseases. Studies indicate that TSPAN4, a migrasome marker, is significantly upregulated in myocardial infarction mouse models and associated with pathological processes such as macrophage enrichment and intra-plaque hemorrhage,^[16]^ suggesting migrasome involvement in atherosclerosis. Targeting TSPAN4-mediated migrasome formation may thus provide a novel therapeutic strategy for atherosclerosis. Zhang *et al*. further demonstrated that endothelial cell-derived migrasomes promote macrophage polarization toward the pro-inflammatory M1 phenotype, accelerating atherosclerosis.^[17]^ Migrasome expression positively correlates with disease severity in advanced-stage patients.^[17]^ Thus, targeting TSPAN4 and migrasome pathways may slow atherosclerosis progression.

Sun *et al*. used a mouse model of myocardial ischemia-reperfusion injury (MIRI) to demonstrate that low-intensity pulsed ultrasound (LIPUS) alleviates MIRI-induced mitochondrial dysfunction.^[85]^ Mechanistically, LIPUS activates the RhoA/myosin II/F-actin signaling pathway, inducing migrasome formation to expel damaged mitochondria. This process mitigates mitochondrial dysfunction, thereby improving cardiac function.^[85]^ Thus, migrasome-mediated mitochondrial quality control exerts cardioprotective effects in MIRI, providing novel avenues for non-invasive cardiac protection strategies. Zhu *et al*. combined machine learning with single-cell RNA sequencing, Mendelian randomization, and molecular docking techniques to develop migrasome-related signatures predicting acute myocardial infarction risk.^[86]^ They identified a significant positive causal relationship between migrasome-related gene ITGB1 expression and acute myocardial infarction risk.^[86]^ Migrasome-mediated mitochondrial quality control may sustain cardiovascular mitochondrial homeostasis, suggesting potential therapeutic strategies for cardiovascular diseases.^[87]^ However, clear *in vivo* evidence remains lacking, and further research is needed.

### Regenerative medicine

Migrasomes exhibit unique biological functions in regenerative medicine.^[19]^ Studies show that adipose‑derived stem cells (ASCs) establish a chemotactic gradient by secreting CXCL12‑rich migrasomes, which activate the CXCR4/RhoA signaling axis to drive stem cell migration and create a pro‑regenerative microenvironment. This process significantly improves soft tissue regeneration efficiency.^[19]^ These findings indicate that ASC‑derived migrasomes restore tissue regeneration by recruiting stem cells. As a novel therapeutic target in ASC‑mediated regeneration, migrasomes offer potential intervention strategies for regenerative medicine. In bone regeneration, Li *et al*. confirmed that M2 macrophage‑derived migrasomes carry osteogenic differentiation‑inducing factors.^[88]^ Migrasomes collected from M2 macrophages using a titanium dioxide nanotube surface enhance the osteogenic differentiation potential of mesenchymal stem cells (MSCs), thereby promoting new bone formation in defect models. Coating titanium surfaces with migrasomes further enhances osteogenesis.^[88]^ Thus, nano‑surfaces may function as platforms for migrasome production, offering new approaches for tissue regeneration.

### Wound healing

Migrasome biogenesis declines markedly during skin aging. Migrasomes derived from young fibroblasts reverse senescent phenotypes in aged keratinocytes and accelerate wound healing in aged skin.^[18]^ Single‑cell RNA sequencing (scRNA‑seq) reveals peak transcriptional heterogeneity in fibroblasts during aging, with senescence levels inversely correlating with migrasome marker expression. Multiplex immunohistochemistry (mIHC) and TEM confirm significantly higher migrasome density in skin samples from young donors (mice/humans) compared with aged counterparts.^[18]^ In naturally aged mouse models, exogenous young fibroblast‑derived migrasomes accelerate wound closure and suppress senescence‑associated marker expression.^[18]^ As novel intercellular communication vectors, migrasomes exhibit dual therapeutic capacities. They function as natural repair engines that accelerate wound healing and as rejuvenation signal hubs that reverse keratinocyte aging. This strategy enables simultaneous senescent cell clearance and tissue regeneration, offering transformative potential for geriatric chronic wound treatment.

### Isolation and purification of migrasomes

Methods for isolating and purifying migrasomes include differential centrifugation and density gradient centrifugation. These techniques effectively minimize contamination from cell bodies, apoptotic bodies, and exosomes, resulting in purified migrasome fractions. Yu *et al*. adapted extracellular vesicle isolation strategies, combining differential centrifugation with density gradient centrifugation, to enrich migrasomes from biological samples (*e.g*., body fluids or conditioned media).^[89]^ Notably, this protocol uses Optiprep rather than sucrose as the density medium, preserving migrasome structural integrity through optimized isopycnic centrifugation parameters.^[90]^ In this method, cells are digested with 0.25% trypsin [neutralized with 10% fetal bovine serum (FBS)], followed by differential centrifugation: low-speed steps at 1000×*g* for 10 min and 4000×*g* for 20 min (4 ° C) to remove cell debris, and high-speed centrifugation at 20,000×*g* for 60 min (4 °C). Density gradient centrifugation is then employed for further purification.^[39,88]^ Saito *et al*. demonstrated that cell-penetrating peptides (*e.g*., pVEC, R9) and viral fusion peptides (*e.g*., SIV) enhance migrasome biogenesis by promoting cell migration, offering novel strategies for functional enrichment studies.^[91]^ Ma *et al*. developed a pipeline combining hierarchical filtration with ultracentrifugation: initial debris removal using a 0.45-μm filter, migrasome capture *via* reverse membrane extrusion, and enrichment of high-purity migrasome-derived nanoparticles (MDNPs) through ultracentrifugation at 100,000×*g*.^[90]^ Although isolated migrasomes are highly enriched, they are not absolutely pure. Due to this inherent limitation, rigorous quality control is essential before conducting functional studies. Techniques such as Western blotting, mass spectrometry, wheat germ agglutinin (WGA) staining, and electron microscopy are useful for migrasome identification and functional characterization.

### Migrasome Labeling Methods

Early migrasome studies identified key markers (*e.g*., TSPAN4, TSPAN9, integrin α5).^[1]^ Migrasomes can be visualized by labeling these markers with green fluorescent protein or mCherry; however, this approach involves prolonged protocols, high cost, and potential interference with migrasome biogenesis due to protein overexpression.^[1,3,92]^ The WGA dye labeling method rapidly labels cell lines and chicken embryos by binding N-acetylneuraminic acid and N-acetylglucosamine, but its efficacy is limited in zebrafish and mice.^[93]^ Liang *et al*. demonstrated that SMS2 forms SM-enriched microdomains at the leading edge of migrating cells, determining MFSs. Non-toxic lysenin (NT-Lys), which specifically binds membrane SM, effectively labels migrasomes *in vitro*, but its applicability for *in vivo* labeling remains unexplored.^[27]^ Cui *et al*. developed an aggregation-induced emission (AIE)-based near-infrared probe, TTCPy, specifically binding migrasome phospholipids to enable rapid fluorescent labeling.^[93]^ TTCPy permits high-resolution imaging of migrasomes in live cells and CAM, and it supports super-resolution microscopy for enhanced spatial contrast.^[93]^ Additionally, Yang *et al*. established a magnetic bead-based assay combining WGA-coated beads with flow cytometry for efficient migrasome isolation and quantification.^[11]^ This method quantitatively compares migrasome levels in urine samples from healthy donors and nephropathy patients, demonstrating potential applicability in diverse liquid biopsies (*e.g*., cell culture media, serum, urine) and clinical translation.^[11]^ However, due to the extremely low abundance of migrasomes in body fluids (such as blood and urine), false-negative results are very likely to occur in routine tests, which severely limits their clinical application potential as disease biomarkers. To overcome this technical bottleneck, in the future, a microfluidic enrichment platform can be used to efficiently capture and concentrate migrasomes. At the same time, combined with a capture strategy based on specific antibodies or affinity ligands, the sensitivity and reliability of detection can be significantly improved, thereby promoting the practical application of migrasomes in the field of non-invasive diagnosis. Migrasome research depends on dynamic tracing and observation techniques. Therefore, developing more efficient, convenient, and specific methods for migrasome labeling and visualization represents a major ongoing challenge that requires continuous exploration and refinement.

### Morphological observation of migrasomes

Optical and electron microscopy techniques enable the observation, detection, and characterization of migrasomes. As early as 1963, Taylor *et al*. used TEM to observe contractile filaments produced by migrating cells.^[94]^ Correlative light-electron microscopy (CLEM) has revealed migrasomes as vesicle-like structures enclosed by a single-layer membrane containing multiple smaller vesicles.^[1]^ Researchers first successfully observed migrasome formation in living cells using confocal microscopy-based live-cell imaging technology.^[3]^ Developing an *in vivo* imaging system to monitor migrasome dynamics in mice is a current research priority. However, traditional spinning-disk confocal microscopy provides insufficient spatiotemporal resolution and higher phototoxicity, limiting its application in migrasome research.

Jiang *et al*. used fluorescently conjugated antibodies combined with flow cytometry to specifically label cell-surface proteins.^[39]^ This method rapidly labels migrasomes in real-time, enabling *in vivo* observation in live mice and facilitating exploration of migrasome functions in physiological processes. Yu Li *et al*., in collaboration with Prof. Dai Qionghai’s team at Tsinghua University, developed Digital Adaptive Optics Scanning Light-field Mutual Iterative Tomography (DAOSLIMIT) technology.^[95]^ Using a scanning light-field microscope, they observed migrasome production by neutrophils and tumor cells at enhanced spatiotemporal resolution through a hepatic observation window.^[95]^ Recently, Prof. Dai Qionghai’s team developed two-photon synthetic aperture microscopy (2pSAM), employing a needle-like beam for high-speed scanning.^[73]^ This technique reduces cellular phototoxicity and provides superior spatial resolution compared to conventional methods. Applying 2pSAM, they documented migrasome production by neutrophils in the cortex of TBI mice.^[73]^

## Differences between migrasomes and exosomes

**Figure 4:**
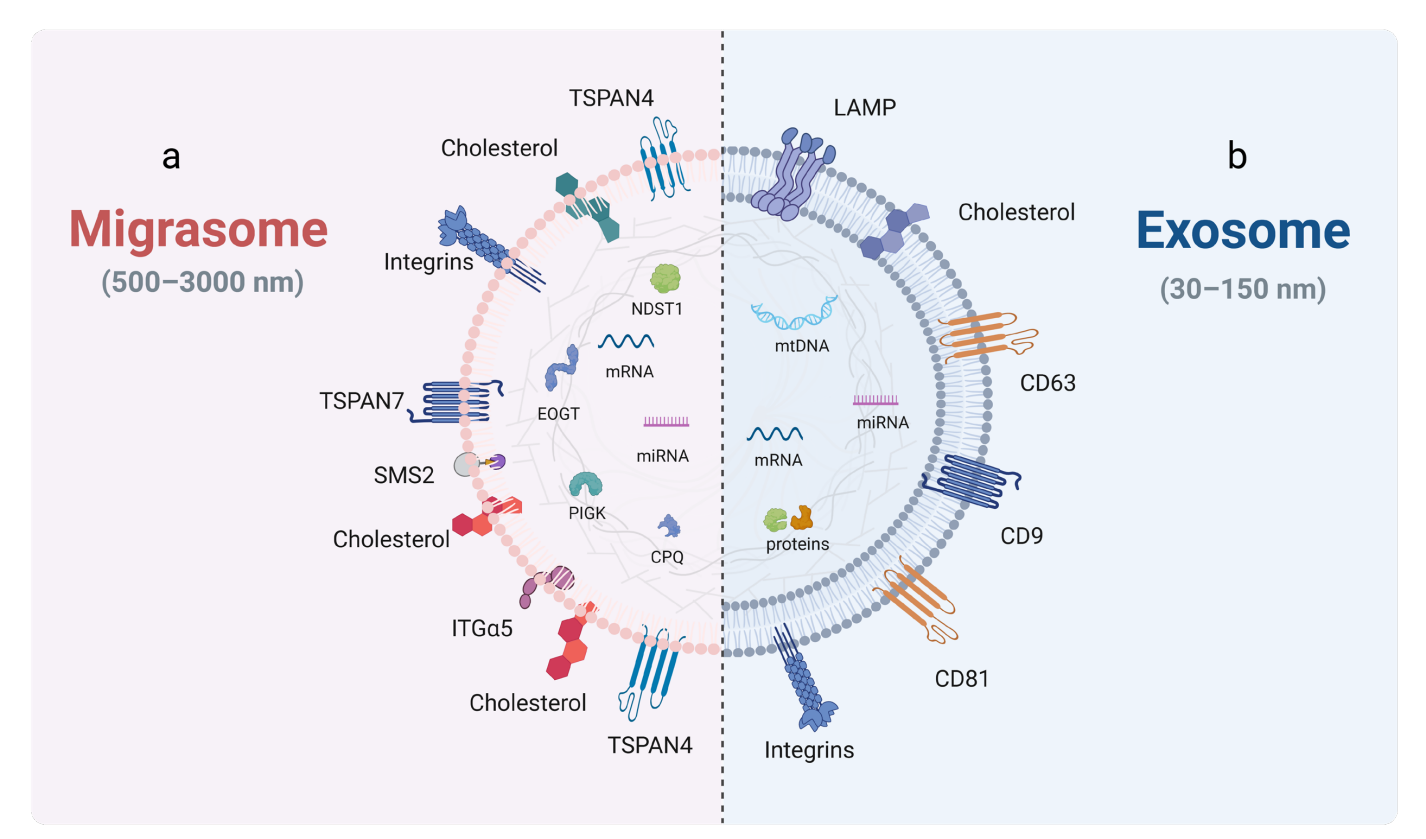
The composition of migrasome and exosome is different. (a) Migrasomes are single-membrane vesicular structures 500–3000 nm in diameter. Their specific markers include NDST1, PIGK, CPQ, and EOGT, and the contents include mRNA, miRNA, *etc*. (b) Exosomes are extracellular vesicles enclosed by a lipid bilayer, with a diameter of 30 to 150 nm. Their specific markers include CD63, CD9, LAMP, *etc*., and the contents include DNA, mRNA, miRNA, ncRNA, *etc*. CD9: cluster of differentiation 9; LAMP: plysosome-associated membrane glycoprotein.

EVs are small membrane-bound vesicles released into the extracellular environment, serving as critical mediators of intercellular communication.^[96,97]^ Although both are vesicular structures, migrasomes differ fundamentally from exosomes (Table 2). Migrasomes perform multiple cellular functions before detaching as EVs; therefore, EV generation represents only one facet of migrasome function.^[98]^ Migrasomes are single-layered, predominantly oval-shaped organelles with diameters ranging from 500 to 3000 nm, containing numerous smaller vesicles (ranging from less than 10 to over 300).^[1]^ Exosomes, however, are lipid-bilayer-enclosed vesicles (30–150 nm in diameter) with distinct protein markers and cargo compositions compared to migrasomes.^[99]^ Exosome-specific markers include CD63, LAMP 1, and LAMP2, whereas migrasome-specific proteins (*e.g*., NDST1, EOGT, PIGK, CPQ) are undetectable in exosomes.^[100,101]^ Exosomal membranes contain lipids and proteins, and their cargo comprises diverse nucleic acids (DNA, mRNAs, miRNAs, ncRNAs) in addition to proteins.^[102]^ Exosomes, secreted by diverse cell types, are abundant in bodily fluids.^[103]^ Migrasomes similarly contain lipids, proteins, mRNAs, and miRNAs. Proteomic analyses reveal only 27% protein identity between migrasomes and exosomes, and sequencing identifies distinct miRNA enrichment profiles (Figure 4).^[10]^

**Table 2:** Differences between migrasomes and exosomes

Charecterize	Biogenesis	Release	Diameter	Markers	Lifecycle	Content	Physiological functions	References
Migrasome	During migration, migrasomes are formed at the tip or bifurcation of the RFs	Breakdown of retraction fibers	500–3000 nm	TSPAN4, TSPAN7, Integrin (α1, α3, α, β1), NDST1, EOGT, PIGK, CPQ and WGA	About 400 min	EVs, impaired mitochondria, mRNA, miRNA, Protein	Maintaining mitochondrial homeostasis; Material transferring; Embryonic development; Angiogenesis; Participate in blood coagulation	[1, 4, 5, 6, 7, 24, 89, 101, 112, 113]
Exosome	Formed *via* the endosomal sorting complexes required for transport (ESCRT)-dependent or ESCRT-independent pathways	MVEs fuse with the plasma membrane	30–150 nm	CD9, CD63, CD81, HSP60, HSP70, HSP90, TSG101, LAMP1, LAMP2	A few minutes to a few hours	DNA, mRNA, miRNA, Ln-cRNA, Protein, Lipid	Intercellular communication and signal transduction; Immune regulation; Removal of waste substances;	[105, 107, 108, 109, 111, 114, 115, 116]

RFs: retraction fibers; ESCRT: endosomal sorting complexes required for transport; TSPAN: tetraspanin; NDST: N-deacetylase and N-sulfotransferase; EOGT: domain-specific O-linked N-acetylglucosamine transferase; PIGK: phosphatidylinositol glycan anchor biosynthesis class K; CPQ: carboxypeptidase Q; WGA: wheat germ agglutinin; MVEs: multivesicular endosomes; CD: cluster of differentiation; HSP: heat shock protein; TSG: tumor susceptibility gene; LAMP: lysosome-associated membrane glycoprotein; DNA: deoxyribonucleic acid; RNA: ribonucleic acid.

**Figure 5:**
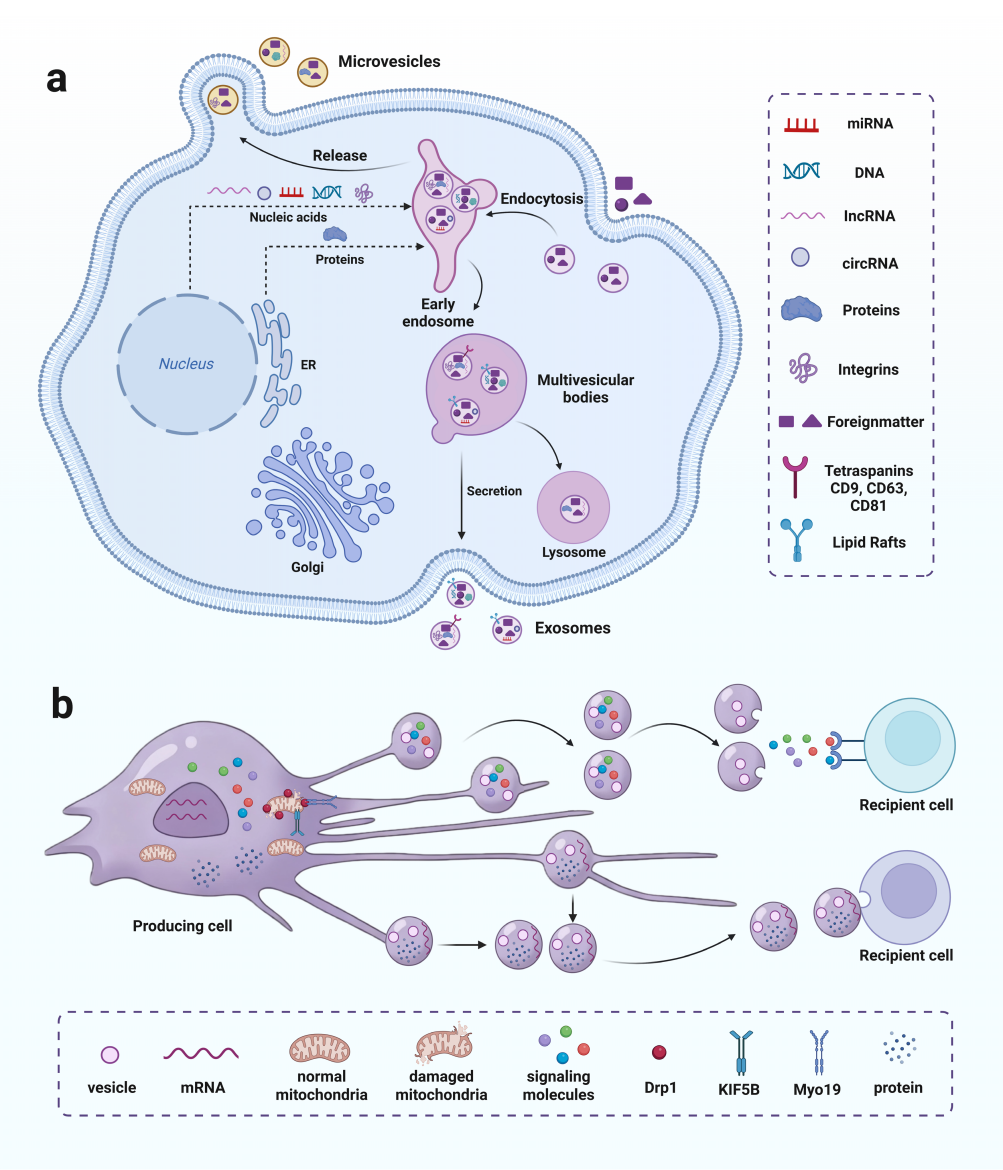
The biogenesis and release of migrasomes and exosomes are different. (a) Exosomes are derived from the endocytosis of the plasma membrane. The plasma membrane invaginates to form early endosomes, which selectively incorporate nucleic acids, proteins, lipids, and other cytoplasmic components, subsequently maturing into multivesicular bodies. The multivesicular bodies then fuse with the plasma membrane, releasing their intraluminal vesicles into the extracellular matrix as exosomes. (b) Migrasomes are generated during continuous cell migration. As retraction fibers break, the mature migrasomes detach from the parent cells and are released into the microenvironment. They mediate intercellular communication either by releasing their contents or by being taken up by neighboring cells.

Biosynthetic pathways of migrasomes and exosomes differ fundamentally. Migrasome formation involves three distinct stages: nucleation, maturation, and expansion.^[21,24,27]^ During sustained cell migration, retraction fibers gradually break, allowing mature migrasomes to detach from the parent cell and enter the extracellular environment. Released migrasomes facilitate intercellular communication through diffusion of their contents or uptake by neighboring cells.^[1]^ In contrast, exosomes originate from the endolysosomal pathway.^[104]^ The plasma membrane invaginates to form early endosomes (EEs). Through further inward budding of the endosomal membrane, multiple intraluminal vesicles (ILVs) form within EEs. After selectively incorporating nucleic acids, proteins, and lipids from the cytoplasm, EEs mature into multivesicular bodies (MVBs). Fusion of MVBs with the plasma membrane releases exosomes into the ECM (Figure 5).^[105,106]^ Functionally, migrasomes and exosomes have distinct roles. Migrasomes regulate cellular homeostasis,^[6]^ mediate intercellular communication,^[3,5]^ and participate in coagulation.^[7]^ Beyond mediating intercellular communication and signal transduction, exosomes facilitate the clearance of damage-induced cellular waste to maintain homeostasis. They also participate in immune regulation, with their functions being highly dependent on their parental cell types.^[105, 107, 108, 109, 110, 111]^

## Conclusion and future perspectives

The scientific definition of migrasomes is a dynamic, evolving concept. Initially, due to their precise membrane structure and topological similarity to cilia, migrasomes were classified as a new type of organelle.^[1,117]^ However, later studies revealed their active detachment from the parent cell and release into the extracellular space.^[7]^ This characteristic endowed migrasomes with core features of EVs, prompting re-evaluation of their classification. Subsequent research has further complicated rather than clarified this debate, highlighting a sophisticated dual-functional nature of migrasomes. Recent evidence indicates that migrasomes, while attached to cells *via* retraction fibers, serve as localized platforms for exocytosis, facilitating directional secretion of proteins.^[50]^ This supports a context-dependent duality: migrasomes function as “subcellular functional domains” dedicated to secretion when attached to cells, and become “autonomous membranous vesicles” upon detachment, mediating intercellular communication. This dual-life-cycle characteristic challenges rigid classification schemes and underscores the need for a more integrative definition. Therefore, describing migrasomes as specialized organelles involved in intercellular communication may offer a more comprehensive perspective.^[118]^ Such a definition accommodates both phases: the “organelle” phase emphasizes active biogenesis and secretory roles on the cell surface, while the “intercellular communication” phase highlights their role as large EVs after release.

As emerging structures in intercellular communication, migrasomes exhibit significant functional heterogeneity and environmental dependence. Their functions depend on molecular cargo, spatiotemporal dynamics, and microenvironmental conditions,^[50,118]^ similar to the functional plasticity observed in autophagy. Studies suggest migrasomes participate in directional transmission of morphogenetic signals during embryonic development and primarily mediate inflammatory or chemotactic factor release in immune regulation. These observations indicate potential migrasome subtypes. However, current evidence for systematic identification of migrasome subpopulations remains insufficient. Integration of advanced technologies, including single-cell sequencing, super-resolution imaging, and specific labeling methods, is required. These approaches could clarify relationships between migrasome molecular composition and function under various physiological and pathological contexts, providing experimental evidence for classification and regulatory mechanisms of migrasome subtypes. Migrasomes are highly heterogeneous due to different cell sources, which makes them both a highly potential specific diagnostic and therapeutic tool and poses a core challenge to the controllability and reliability in their clinical translation.

With advancing research into migrasome functions, their clinical relevance has attracted increasing attention. During viral infection, migrasomes encapsulate viral particles, promoting transmission and contributing to drug evasion;^[15,55]^ they may also facilitate tumor cell metastasis and invasion.^[8,56]^ Consequently, targeting migrasomes represents a potential therapeutic strategy, as reducing their abundance could improve disease prognosis. Migrasomes also exhibit beneficial roles in tissue regeneration and wound healing;^[18,19]^ thus, identifying substances that promote migrasome production may help compensate for deficiencies under pathological conditions. However, migrasome roles in disease mechanisms remain incompletely understood. For instance, in CAA, migrasomes have been implicated in pathology. Yet, inhibiting migrasomes might trigger compensatory release of molecules (*e.g*., CD5L, Aβ) through other EVs such as exosomes or microvesicles from macrophages, potentially exacerbating disease progression. In urinary diseases, podocyte-derived migrasomes in urine could serve as early biomarkers of renal injury. Nevertheless, compared to established clinical indicators (*e.g*., eGFR, UACR), the reliability and clinical applicability of migrasomes require further validation. Currently, evidence supporting clinical use of migrasomes remains insufficient. Developing diverse animal disease models is thus necessary to systematically investigate migrasome dynamics across pathophysiological contexts, enhancing their sensitivity and specificity as biomarkers. Additionally, given their efficient intercellular transport capability, migrasomes hold promise as targeted drug delivery vehicles.

Early migrasome studies utilized models suitable for direct microscopic imaging, including zebrafish embryos and the chick CAM.^[4,23]^ Recent advances in mouse models now enable *in vivo* migrasome observation. However, research still relies heavily on *in vitro* systems and zebrafish. Although valuable for mechanistic studies, these models do not fully replicate the complex physiological or pathological environments or dynamic regulatory networks in mammals. This limitation restricts deeper insights into migrasome functions and disease relevance. Therefore, systematically examining migrasome functions *in vivo*, especially in disease contexts, is urgently needed. A recommended approach involves genetically engineered mouse models (*e.g*., conditional knockout or knockin of migrasome-related genes) combined with established disease models such as tumor xenografts or neurodegenerative disorders. This strategy would clarify migrasome regulation of disease processes in whole organisms. Humanized models, such as patient-derived organoids or xenografts, could further replicate human-specific genetics and microenvironments, providing clinically relevant platforms. In parallel, isolating migrasomes from clinical samples (*e.g*., blood, cerebrospinal fluid) and analyzing their composition and quantity relative to disease stage, treatment response, and prognosis could establish a translational foundation for migrasome research.

Significant progress has been made in understanding the biogenesis, physiological functions, and pathological roles of migrasomes. However, migrasome research remains in early stages compared to exosomes, with many key aspects still unclear. As membrane-bound vesicles, migrasome half-life is influenced by structural stability, extracellular environments, and clearance mechanisms. Studies show migrasomes can deliver signaling molecules to inflammatory sites *via* circulation, highlighting their potential as natural drug delivery platforms.^[119]^ However, their *in vivo* half-life and targeting efficiency require further investigation. Although migrasomes, as endogenous vesicles, theoretically possess low immunogenicity, emerging evidence indicates they may facilitate signal transmission and inflammation under certain pathological conditions.^[74]^ Therefore, detailed mechanisms underlying their immunomodulatory effects and associated risks require comprehensive evaluation.

Understanding migrasome biogenesis and their physiological and pathological roles remains challenging. A primary issue is the lack of standardized methods for migrasome isolation and purification. Current methods primarily involve ultracentrifugation and density gradient centrifugation.^[90,120]^ However, migrasomes have low abundance in body fluids (*e.g*., blood) and often co-precipitate with cell debris, resulting in insufficient purity. Existing isolation techniques, although feasible, frequently require large sample volumes and complex procedures. Furthermore, the resulting product is a highly enriched migrasome fraction rather than absolutely pure migrasomes, complicating downstream research. Additionally, migrasome quantification methods vary in sensitivity, reducing data comparability across studies. Thus, efficient migrasome isolation and purification remain key bottlenecks in both basic research and clinical applications. Advanced technologies (*e.g*., microfluidics, automated systems) should be adopted for visualization, biochemical characterization, and quantitative detection of migrasomes. Establishing robust and reproducible protocols for migrasome isolation and detection will facilitate their diagnostic and therapeutic applications.

Technical limitations significantly hinder progress in migrasome research. Besides improving isolation and purification methods, there is an urgent need to establish techniques for dynamically tracking migrasomes *in vivo*. A primary challenge is achieving high spatiotemporal resolution and prolonged observation within complex biological environments. Current approaches mainly combine high-resolution live imaging with specific labeling. These methods are effective in transparent systems such as zebrafish embryos but are limited in mammals due to poor tissue penetration and phototoxicity. Currently, only short-term tracking in transparent or superficial tissues is feasible. Moving forward, integrating AI-assisted image analysis with multi-omics technologies will be essential to accurately characterize migrasome functions *in vivo*. With continued collaborative efforts, developing a comprehensive spatiotemporal atlas of migrasome biogenesis is achievable. Such advancements will greatly enhance our understanding of migrasome functions in physiological and pathological contexts and clarify their broader biological significance.
